# Application of time-frequency domain and deep learning fusion feature in non-invasive diagnosis of congenital heart disease-related pulmonary arterial hypertension

**DOI:** 10.1016/j.mex.2023.102032

**Published:** 2023-01-20

**Authors:** Pengyue Ma, Bingbing Ge, Hongbo Yang, Tao Guo, Jiahua Pan, Weilian Wang

**Affiliations:** Yunnan University, Fuwai Yunnan Cardiovascular Hospital, China

**Keywords:** Time-frequency domain features, Power-normalized cepstral coefficients, Convolution neural network, XGBoost, Pulmonary arterial hypertension, Fusion of time-frequency domain features and depth features and classification of XGBoost.

## Abstract

Pulmonary arterial hypertension associated with congenital heart disease (CHD-PAH) is a fatal cardiovascular disease. A novel method for non-invasive initial diagnosis of the CHD-PAH was put forward in this work. First, original heart sounds were segmented into each cardiac cycle by using double-threshold adaptive method. According to clinical auscultation, the pathological information of CHD-PAH is concentrated in S2, so the time-frequency features in both of an entire cardiac cycle and S2 were extracted. Then the time-frequency features combine with the deep learning features to form a feature vector. It is the fusion feature, which will be input into a classifier. Finally, the majority voting algorithm was used to obtain the optimal classification results. A classification accuracy of 88.61% was achieved using this novel method. Three points are essential:

• A double-threshold adaptive method is used to segment heart sound into each cardiac cycle.
• The time-frequency domain features in both of an entire cardiac cycle and S2 were extracted, which are combined with deep learning features to form the fusion feature.
• The XGBoost was used as three-class classifier for the classification of normal, CHD and CHD-PAH. The majority voting algorithm was used to obtain the optimal classification results.

## Graphical abstract

The overall diagram

**Figure:**
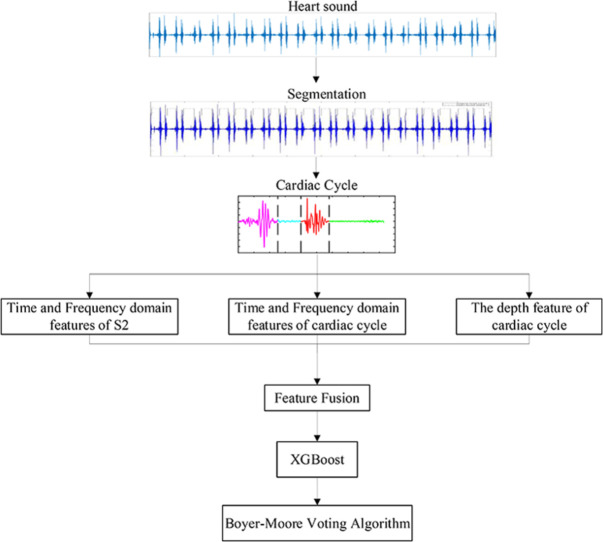

Specifications table

**Table:** 

Subject area:	Medicine and Dentistry
More specific subject area:	Biomedical engineering
Name of your method:	Fusion of time-frequency domain features and depth features and classification of XGBoost.
Name and reference of original method:	1. MFCC, CNN Kui H, Pan J, Zong R, Yang H, Wang W. Heart sound classification based on log Mel-frequency spectral coefficients features and convolutional neural networks. Biomedical Signal Processing and Control, 2021, 69. 2. Adaptive segmentation of breath sounds based on S-transform threshold Chen, Hai; Yuan, Xiaochen; Li, Jianqing; Pei, Zhiyuan; Zheng, Xiaobin. Automatic Multi-Level In-Exhale Segmentation and Enhanced Generalized S-Transform for Wheezing Detection. Computer Methods and Programs in Biomedicine, 2019. 3. Multi-domain feature fusion Li H, Wang X, Liu C, Zeng Q, Zheng Y, Chu X, Yao L, Wang J, Jiao Y, Karmakar C. A fusion framework based on multi-domain features and deep learning features of phonocardiogram for coronary artery disease detection. Comput Biol Med. 2020 May. 4. PNCC C. Kim and R. M. Stern.Power-Normalized Cepstral Coefficients (PNCC) for Robust Speech Recognition. IEEE/ACM Transactions on Audio, Speech, and Language Processing, July 2016,vol. 24, no. 7, 1315–1329. 5. Classification of Heart Sounds Based on Time Domain Features Elgendi M, Bobhate P. Time-domain analysis of heart sound intensity in children with and without pulmonary artery hypertension: a pilot study using a digital stethoscope. Pulm Circ. 2014 Dec, 685–95.
**Resource availability**	hardware {CPU: Intel Core i7 @4.4 GHz Memory:16GB Graphics card: NVIDIA 3060 6GB} software {python3.7.5 tensorflow2.6.0 matlab2021}

**Method details**

## Signal preprocessing

**Fig. 1:**
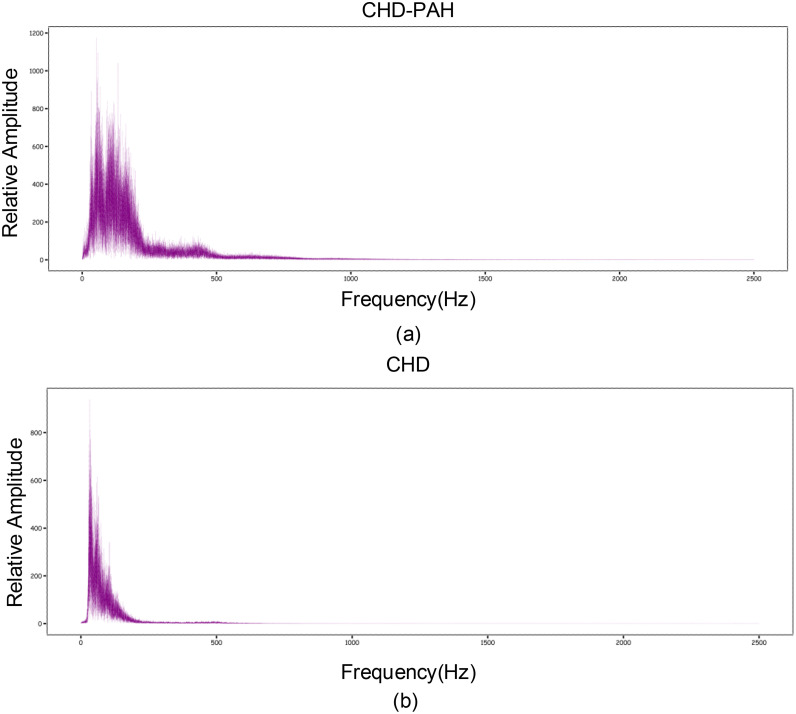
Spectrogram of heart sound.

Data preprocessing is the first step to achieve the purpose of heart sound classification. Here, the original sampling frequency of heart sounds is 5000 Hz. Fourier transform the heart sound and draw the heart sound spectrogram. As shown in Fig. 1 the heart sound signal below 1000 Hz contains all the key information about CHD-PAH and no any useful information is lost when down sampling. The heart sounds were down sampled to 2500 Hz and then segmented. Because the heartbeat is an infinitely cyclic process, the heart sounds made by the systolic and diastolic attempts also exhibit quasi-periodic properties. A cardiac cycle is the complete process of a heartbeat, and it is also the smallest unit of heart sound.

## Segmentation of heart sound signal

A piece of original heart sound signal has a length of 20 s, and each S1, systolic, S2, and diastolic period it contains is labeled. Then, according to the needs of the experiment, the complete cardiac cycle and the corresponding S2 component are saved separately, and the heart sound segmentation process of this experiment is completed. This paper adopts the method of adaptively segmenting S1/S2 by detecting endpoints by threshold. In the heart sound signal, the energy of S1 and S2 is much higher than the energy of systole and diastole. If the heartbeat contains a distinct human voice, then it will have a larger spectral center of mass because the heartbeat signal tends to have a lower frequency spectrum and therefore a smaller spectral center of mass to filter out some of the murmurs in the HS.Thus the S1/S2 segment, diastolic/systolic segment can be segmented using short time energy and spectral mass cores.

**Fig. 2:**
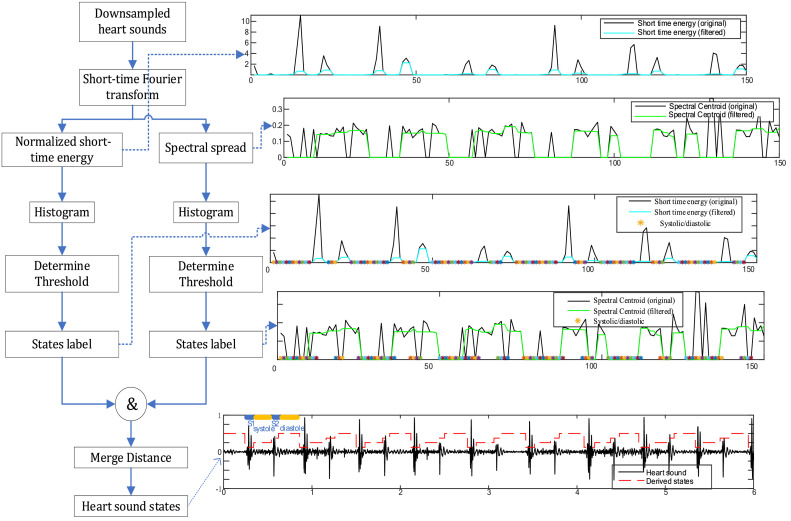
Flowchart of Heart sound segmentation.

The specific flowchart is shown in Fig. 2. The segmentation algorithm extracts the short-term energy and spectral dispersion calculated envelopes from each frame, which can well weaken the influence of low-value noise pairs, making low-intensity sounds easier to find. Then create histograms of short-term energy and spectral spread distributions. For each histogram, a threshold is adaptively determined. Perform AND operation on the segmentation results of the two thresholds, and finally obtain the labeled heart sound signal.

The specific steps of heart sound segmentation processing are as follows:

1) Convert the heart sound signal to a time-frequency representation using the short-time Fourier transform. The heart sound is divided into frames using the Hamming window, the specified window is 0.1 s and the heart sound signal is divided into several sample frames without overlapping.
  (1) $$STFT\left(t,f\right)=\int_{-\infty }^{\infty }x\left(\tau \right)\omega \left(\tau -t\right)e^{-j2\pi f\tau }d\tau$$

where ω(t) is the window function, that is, the heart sound signal x[t] is multiplied by a window function centered at t at time t, and then Fourier transform is performed.

2) Extract two feature sequences. The short-term energy$\;E_{i}$ and the spectral spread $S_{i}\;$are calculated for each frame.
  (2) $$E_{i}=\frac{1}{N}\sum_{n=1}^{N}{\left|x_{i}\left(n\right)\right|}^{2}$$

where $x_{i}\left(n\right)$, *n* = 1,⋯, N is the i−th frame heart sound, and its length is N.

(3) $$S_{i}=\sqrt{\frac{\sum_{i=b_{1}}^{b_{2}}{\left(f_{i}-\mu_{1}\right)}^{2}s_{i}}{\sum_{i=b_{1}}^{b_{2}}s_{i}}}$$

where$\;f_{i}$ is the frequency corresponding to the i−th frame, in Hz; $s_{i\;}$is the spectral value of the i−th frame; $b_{1}\;$and $b_{2}$ are the boundary values ​​of the i−th frame, which are used to calculate the spectral spread. $\mu_{1\;}$is the spectral center point, calculated according to the description of the spectral center point function.

3) Calculate the histograms of the above two feature sequences respectively.
4) For the histogram of each sequence, dynamically estimate two thresholds $T_{E}\;$and $T_{S\;}$according to E formula 4.
  (4) $$T=\frac{W\cdot M_{1}+M_{2}}{W+1}$$

where $M_{1}\;$and$\;M_{2}\;$are the positions of the first and second maximum values, respectively, and W is a fixed value.

5) A simple threshold criterion is used for the sequence. If$\;E>=T_{E}$ is satisfied, it is recorded as flag1, and if $S>=T_{S}$ is satisfied, it is recorded as flag2, which are all suspected heart sound components. The distance of each adjacent candidate is calculated by formula 5.
  (5) $$Dist_{x|x\in \left\{flags\right\}}\left(flag_{x_{-}i},flag_{x_{-}i+1}\right)=\frac{\left|flag_{x_{-}i+1}-flag_{x_{-}i}\right|}{F_{s}}$$

where Fs is the sampling rate. If the distance between segments labeled as suspected heart sound components is less than the merge distance, these segments will be merged. In this experiment, the criterion for the merging distance was set to 50 ms. At this time, those that satisfy the conditions are determined as S1/S2 components. After the thresholding process in the previous step, obtain the threshold sequence of the marked heart segments, which are then mapped to the original signal to obtain the start and end positions of the S1/S2 segments in the original signal. To identify heart sounds based on physiological parameters, the time interval between all adjacent components was calculated. Because diastole is the maximum time interval between two adjacent components (from the end of S2 to the beginning of S1). The corresponding start object is marked as S2, the end object is marked as S1, and then each segment is marked forward and backward in turn. The flowchart is shown in Fig. 2.

One cardiac cycle represents a complete heartbeat, and the feature extraction of heart sounds is performed in this unit. It not only regulates that all heart sound signals have the same start state and end state, but also expands limited heart sound samples and extracts pathological features more accurately and efficiently. In the subsequent majority voting process, multiple results can be predicted for multiple cardiac cycles under the same case, which improves the overall accuracy of the algorithm and reduces the misjudgment of results by chance.

## Feature extraction

For the specific situation of limited samples, a fusion feature including time domain features, frequency domain features, and deep features obtained by deep learning is proposed in feature extraction. Not only the time-frequency domain features of the entire cardiac cycle will be extracted by us, but the time-frequency domain features of S2 will also be extracted. Because clinical auscultation found that the pathological features of congenital heart disease-related pulmonary arterial hypertension were concentrated in S2.

### Time domain features

The original heart sound signal is a one-dimensional time-domain signal, which is usually diagnosed by distinguishing subtle differences in pitch and time during auscultation. Clinicians also focus their attention on each phase of each cardiac cycle in turn, paying attention to every sound and murmur of the heartbeat. Therefore, analyzing the intensity, pitch, duration and time interval of the sound can provide effective feature information for the classification of heart sounds.

Here, 9 time domain features are selected. These characteristics are described in Table 1. Two of the features are about the cardiac cycle. 5 features are related to S2 and 2 features are related to the ratio of cardiac cycle and S2. On auscultation, the heart sounds of CHD-PAH are characterized by hyperactivity and splitting of the S2 component. Here, we introduce the phase value corresponding to the largest value in the cardiac cycle and the S2 component, the phase value corresponding to the second largest value, and the corresponding phase difference as classification features. Select the cardiac cycle and S2 component from the labeled heart sounds, and use formula 6 to calculate and extract the corresponding heart sound intensity as a feature.

(6) $$I_{k}=\sum_{i=1}^{n}{\left(x_{k}\left(i\right)\right)}^{2}$$

where k is the cardiac cycle (cc) or S2. n is the total number of extracted event center tone samples. Heart sound intensity can often reflect the filling of the atrioventricular, valve activity, myocardial contractility and contraction rate, etc., and has a good classification and reference value for machine diagnosis.

**Table 1:** Time domain feature selection.

No.	Feature	Description
1	$i_{cc},\;i_{S2}$	Intensity of cardiac cycle and S2
2	$I_{ratio}$	Ratio of S2 to cardiac cycle intensity:$i_{s2}/i_{ccy}$
3	$l_{cc},\;l_{s2}$	Interval between cardiac cycle and S2
4	$L_{ratio}$	Ratio of S2 to cardiac cycle interval:$i_{s2}/i_{ccy}$
5	$M_{s2}$	S2 and maximum value of cardiac cycle
6	$M2_{s2}$	S2 and second maximum value of cardiac cycle
7	$pos_{S2}$	Phase difference between maximum and second maximum value in S2

### Frequency domain features

**Fig. 3:**
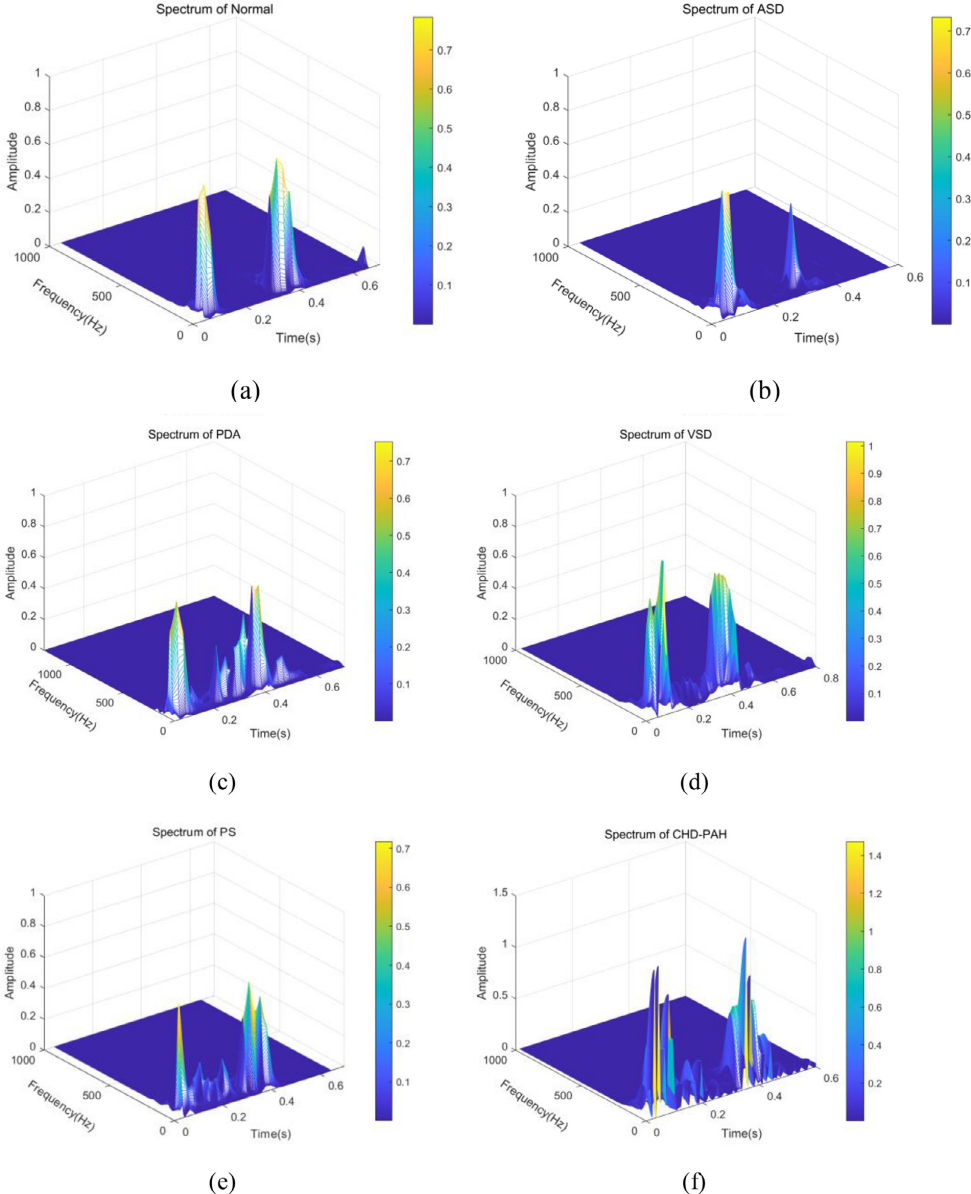
Heart audio spectrogram based on cardiac cycle. (a) Spectrogram of normal heart sound; (b) Spectrogram of heart sound of ventricular septal defect; (c) Spectrogram of heart sound of patent ductus arteriosus; (d) Spectrogram of heart sound of ventricular septal defect; (e) Spectrogram of heart sound of pulmonary artery stenosis Spectrogram; (f) Spectrogram of CHD-PAH heart sound.

Each cardiac cycle of the PCG signal is divided into four states: S1, systole, S2 and diastole. Considering the clinical manifestations in CHD-PAH, the S2 component is often accompanied by hyperactivity and fragmentation. Spectra of some typical cardiac sound signals can be seen in Fig. 3, which have moderate energy distributions during systole and diastole. The complete cardiac cycle and S2 are the focus of this study.

A DFT with a sampling point of 256 points is performed on the cardiac cycle (CC), and a DFT with a sample point of 512 points is performed on the S2. In order to improve the frequency resolution, a simple method is adopted, and a certain number of 0 s are added after the data to make the length reach the required number of points, which can improve the frequency resolution to a certain extent. Afterwards, its spectral features and high-order statistical features are calculated respectively. Because the DFT of real numbers has the property of conjugate symmetry, it retains half the value of its spectrum, and a DC component, that is, the component with frequency zero.

Calculate its corresponding spectral entropy ($es_{cc},es_{s2}$), frequency center point ($C_{cc},C_{s2}$), peak frequency ($pf_{cc},pf_{s2}$), the average value ($af_{cc},af_{s2}$) of fundamental frequency measured in the whole signal, minimum fundamental frequency measured in the entire signal ($minf_{cc},minf_{s2}$), maximum fundamental frequency measured in the entire signal ($maxf_{cc},maxf_{s2}$).

**Fig. 4:**
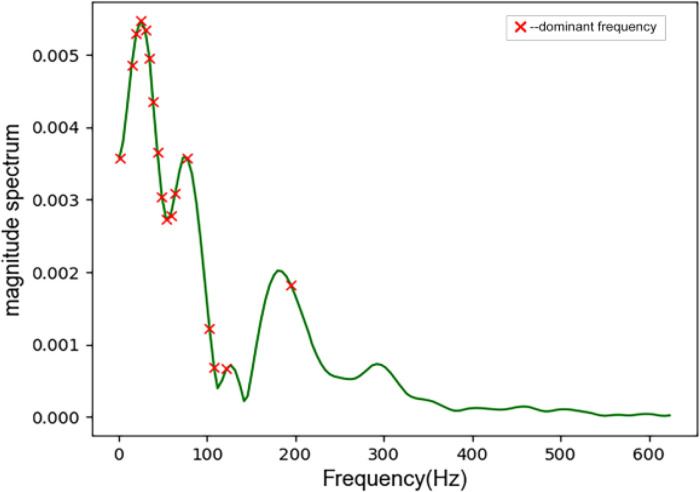
Dominant frequency of cardiac cycle.

**Fig. 5:**
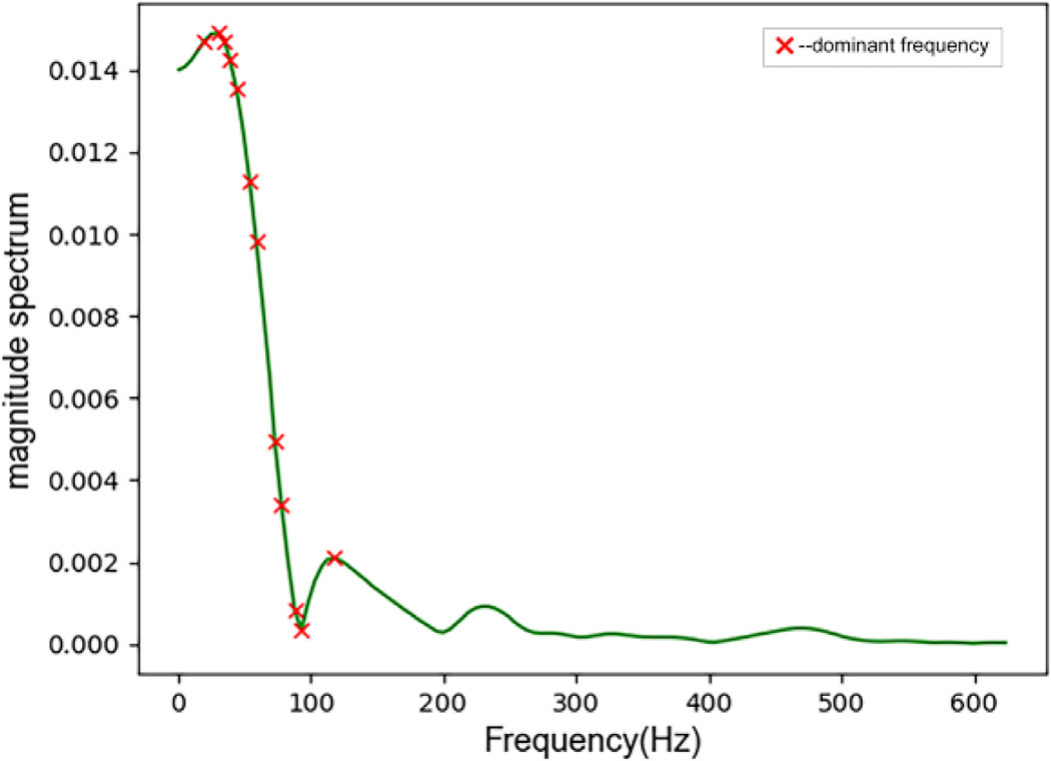
Dominant frequency of the S2 component.

Some peaks in the spectrum correspond to the dominant frequencies, frame the signal, apply the Hamming window function to avoid spectral leakage, find the peaks in the power spectral density of the signal in each frame, get the dominant frequencies, and eliminate the same frequencies at the same time, only keeping unique values, as shown in Figs. 4 and 5. The identification of dominant frequencies helps to understand the structure of time series and to deduce the consequences of the presence and strength of dominant frequencies. Identifying dominant frequencies is part of data analysis, leading to better predictions and more accurate diagnoses.

Extract the average of the dominant frequencies measured in the whole cardiac cycle and the S2 component signal ($ad_{cc},ad_{s2}$), the minimum dominant frequency ($mind_{cc},mind_{s2}$), the maximum dominant frequency (($maxd_{cc},maxd_{s2}$), and dominant frequency range ($dd_{cc},dd_{s2}$) as features.

### The deep learning features

The image obtained by first using the de-differentiated PNCC is used as input for feature learning in the convolutional neural network. Power-normalized cepstral coefficients are a robust recognition feature. It is calculated by following steps:

a) Pre-emphasis

To enhance the high frequency components of the cardiac sound signal, a pre-emphasis filter is used. The pre-emphasis filter H(z) enhances the main elements of the heart sound while successfully suppressing random noise. The filter H(z) is shown in formula 7:

(7) $$H\left(z\right)=1-kz^{-1}$$

where the parameter k is 0.95.

b) Short-time Fourier transform

Time-frequency analysis of the cardiac sound signal of a single cardiac cycle is done by using a short-term Fourier transform. To achieve the same number of output frames, the frame length is adjusted according to the length of each cardiac cycle. It is defined in formula 8:

(8) $$X\left(k\right)=\sum_{n=0}^{N-1}x\left(n\right)e^{-\frac{j2\pi kn}{N}},0\leq n,k\leq N-1$$

where X(k) represents the frequency spectrum of the signal, and x(n) represents the pre-emphasized heart sound signal. N is the number of samples per frame. Because the number of frames is fixed, the length of each frame tends to vary with the length of the cardiac cycle.

c) Power Spectrum

Summing the filtered signals, P(ω) obtains the short-time spectral power, as shown in formula 9:

(9) $$P\left[m,l\right]=\sum_{k=0}^{N-1}{\left|X\left[m,e^{j\omega_{k}}\right]H_{l}\left(e^{j\omega_{k}}\right)\right|}^{2}$$

where m represents the frame, l represents the channel index, $\omega_{k}=2\pi k/Fs$, Fs represents the sampling frequency, and $X[m,e^{j\omega_{k}}]$ is the short-time spectrum of the m-th frame heart sound signal.

$H_{l}\left(e^{j\omega_{k}}\right)\;$represents the $l^{th}$ channel $\omega_{k}$ at the frequency. The formula is shown in 10.

(10) $$\sum_{k=0}^{N-1}{\left|H_{l}\left(e^{j\omega_{k}}\right)\right|}^{2}=1$$

d) Medium time power calculation

By computing the running average of P[m,l], analyzing the measured power from a single frame function, use formula 11 to estimate a quantity we designate as $\tilde{Q}\left(m,l\right)$ during processing using formula 11:

(11) $$\tilde{Q}\left(m,l\right)=\frac{1}{2M+1}\sum_{m^{\prime }=m-M}^{m+M}p\left(m^{\prime },l\right)$$

where l, m and M represent the number of filtering channels, heart sound frames and smoothing window frames, respectively, and $p(m^{\prime },l)$ is the short-term power spectrum of the heart sound signal. Here, the M parameter is set to 2.

e) Asymmetric noise suppression

**Fig. 6:**
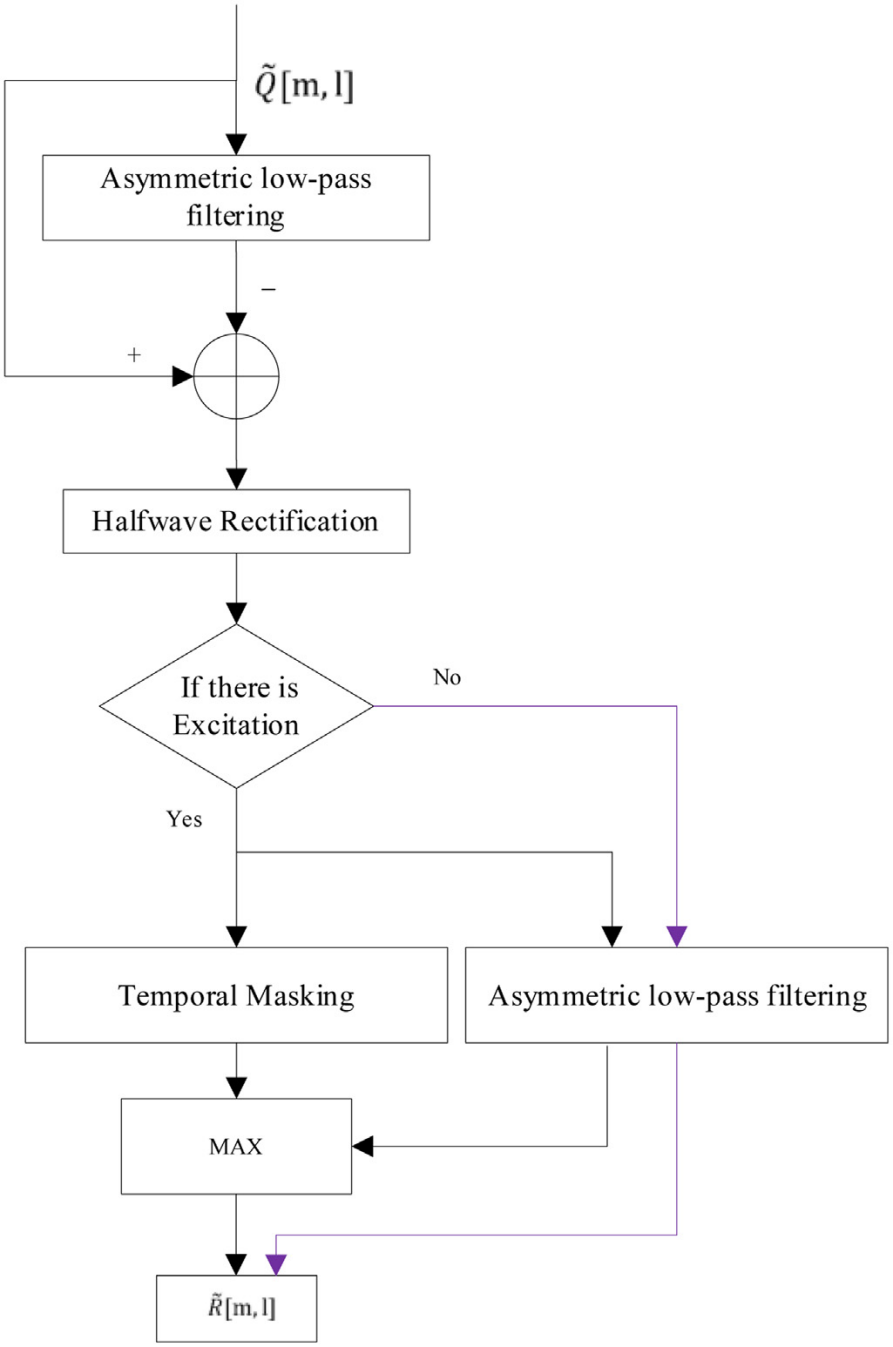
Cal$\text{culate}\;\tilde{R}\left[m,\;l\right].$

Since the energy of the heart sound changes faster than the noise, the high frequency part can be filtered out by spectral subtraction to achieve the purpose of suppressing the noise. The calculation is carried out through the flowchart shown in Fig. 6.

f) Spectral weight smoothing

The transfer function can be used to account for the combined effect of asymmetric noise suppression and temporal masking at a specific time frame and R[m,l]/Q[m,l] band. Smoothing transfer function over frequency is done by finding the running average of the ratios across the channel indices. The frequency average weighting function can be written as shown in Eq. (12):

(12) $$\tilde{S}\left[m,l\right]=\left(\frac{1}{l_{2}-l_{1}+1}\sum_{l^{\prime }=l_{1}}^{l_{2}}\frac{\tilde{R}\left[m,l^{\prime }\right]}{\tilde{Q}\left[m,l^{\prime }\right]}\right)$$

where$\;l_{1}=max\left(l-N,\;1\right),l_{2}=min\left(l+N,\;L\right)$, L is the total number of channels. The initial short-term power P[m,l] is modulated with a time-averaged, frequency-averaged transfer function $\tilde{S}\left[m,l\right]$. The formula for the frequency averaging weighting function is shown in formula 13:

(13) T[m,l]=P[m,l]S[m,l]

g) Mean power normalization

Normalizes the input power by dividing it by an average of the total power. The average power estimate μ[m] is calculated from formula14:

(14) $$\mu \left[m\right]=\lambda_{\mu }\mu \left[m-1\right]+\frac{\left(1-\lambda_{\mu }\right)}{L}\sum_{l=0}^{L-1}T\left[m,l\right]$$

where m and l represent frame and channel indices respectively, and L is the number of frequency channels.The normalized power is calculated from the estimated operating power μ[m] shown in Eq. (15):

(15) $$U\left[m,l\right]=k\frac{T\left[m,l\right]}{\mu \left[m\right]}$$

h) Rate-level nonlinearity

In order to be closer to the pressure sensing characteristics of the human auditory nervous system, PNCC uses an exponential function to be more suitable for the pressure perception of the human auditory nervous system. Its formula is shown in 16:

(16) $$V\left(m,l\right)={\left(k\frac{T\left(m,l\right)}{\mu \left(m\right)}\right)}^{\frac{1}{15}}$$

where l is the order of the filter, k is an arbitrary constant, T(m,l) is the weighting function of the frequency mean obtained by time smoothing, and μ(m)is the mean power.

Deep learning features means that features are extracted by deep learning methods. To extract deep learning features, in the above process, PNCCs are obtained from PCG signals and converted into images. These images are processed by CNN-estimated filters to learn the properties of PNCC images, which help improve classification performance. A convolutional block in a CNN can be thought of as a deep feed-forward network containing convolutional operations, and as an automatic feature extractor. The convolutional layer extracts feature from the input PNCC graph through a convolutional kernel. The pooling layer is located after the convolutional layer and reduces the number of connections between the convolutional layers through pooling operations, and performs downscaling and secondary feature extraction on the PNCC sequence, which together are called convolutional units. Finally, the local features extracted by all convolutional units are aggregated by the fully connected layer, and the depth features. The fully-connected layer with 64 neurons maps high-dimensional deep learning features to low-dimensional features for classification.

**Fig. 7:**
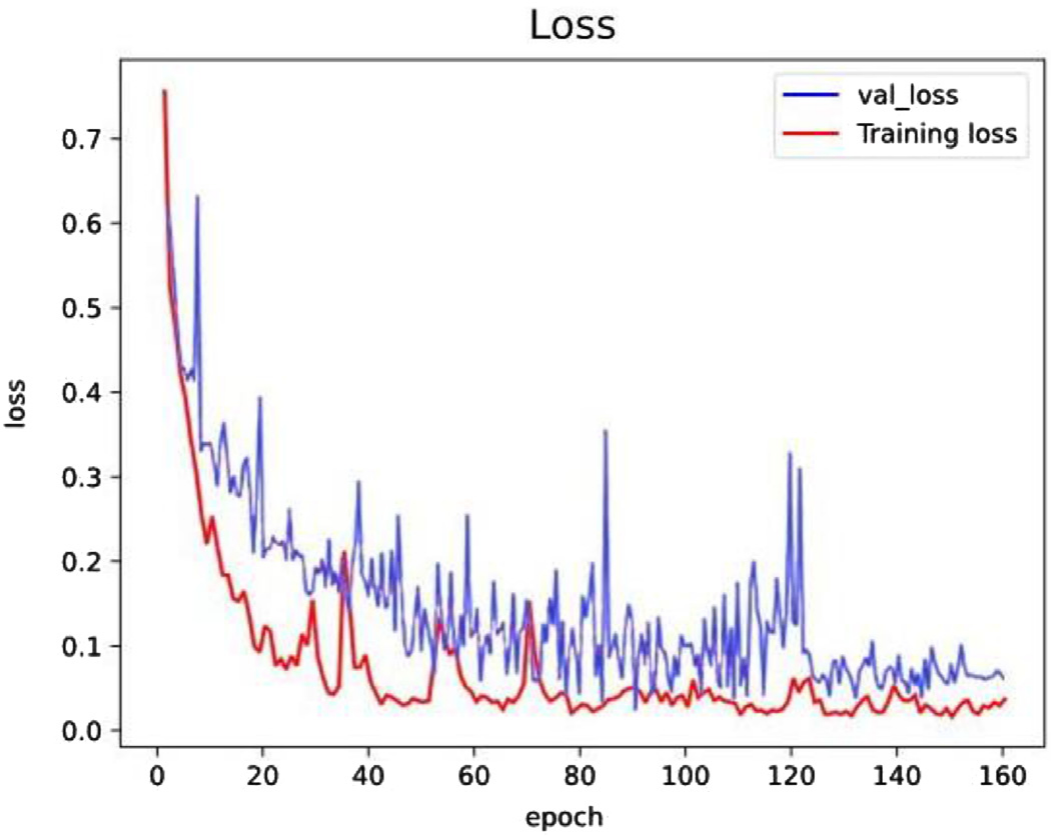
Loss curve.

By setting the Adam hyperparameters to η=0.001, β1=0.9, β2=0.999, adding a dropout parameter to the CNN structure prevents the model from overfitting. In this paper, a CNN model whose loss curve tends to converge is used. The loss curve is shown in Fig. 7.

**Fig. 8:**
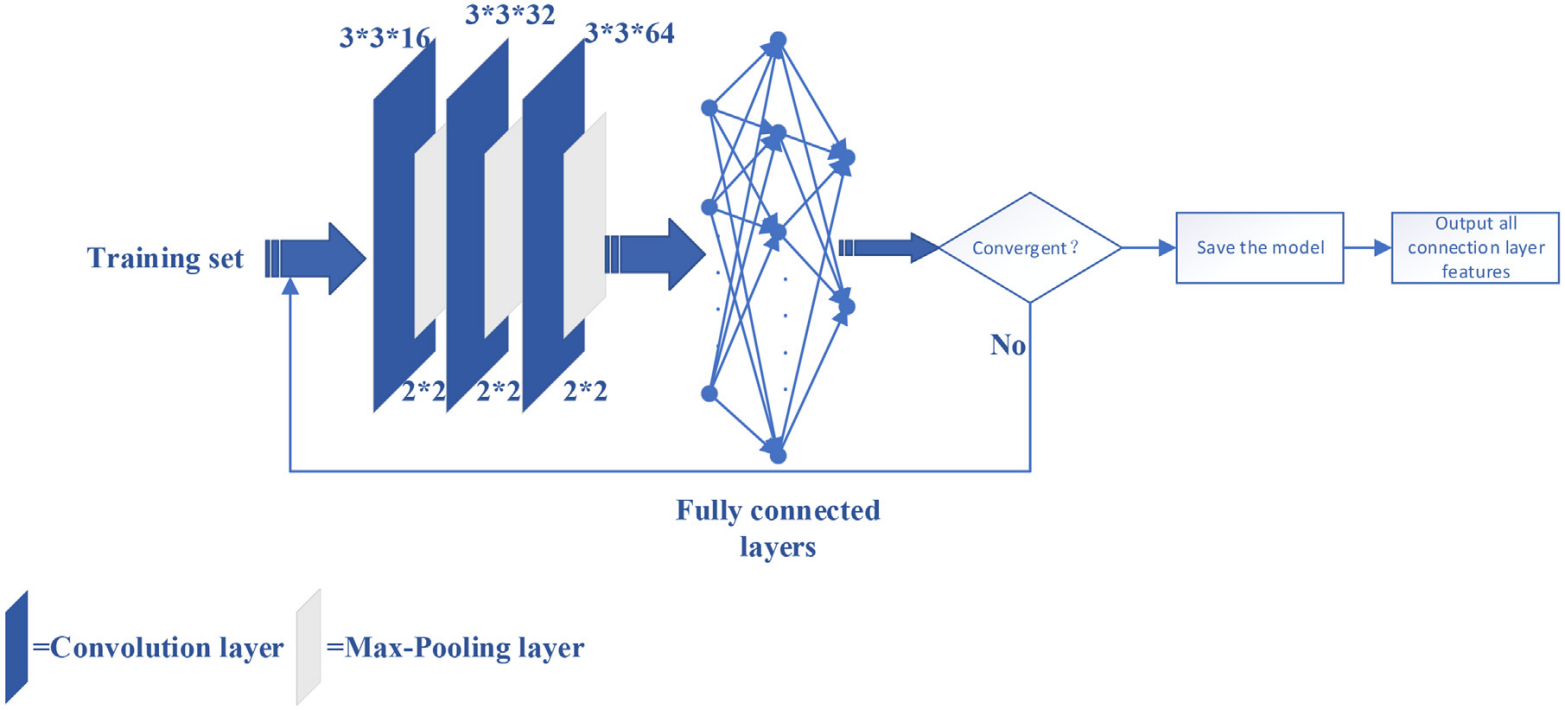
The structure of CNN.

The CNN has the following structure: 1 input layer, 3 convolutional layers, 3 pooling layers, and 1 fully connected layer, as shown in Fig. 8. Save the trained model and substitute the data into the model to obtain feature vectors. In general, to reduce computational complexity, pooling layers usually shrink the convolutional features and extract the main features of the feature map. A fully-connected layer maps the merged features to the example label space; a fully-connected layer with 64 neurons maps high-dimensional deep learning features to low-dimensional features for classification. This study does not directly use CNN as a classifier, but extracts the depth features of the de-discrete PNCC feature image through the CNN fully connected layer, so that the output of the CNN fully connected layer becomes the final retrieval to obtain effective features from the feature map. CNN is also able to extract effective pathological information of CHD-related pulmonary hypertension because of its excellent feature extraction technology.

**Fig. 9:**
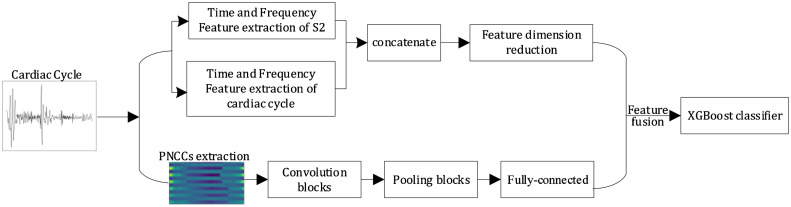
Fusion framework based on PCG time-frequency domain features and deep learning features.

Feature vector based on PCG time-frequency domain feature extraction and deep learning features. First, the heart sound signal was preprocessed. Second, the PNCC and time–frequency domain features were extracted from the preprocessed heart sound signals. Then the time–frequency domain features were optimized, A sequence of features, initially of length 298, was downscaled to a length of 212, in which there are 148 time–frequency domain features and 64 deep learning features. Features with a variance of less than 0.05 are filtered out first, and then the importance score for each feature can be obtained directly after the XGBoost tree model is created. The importance scores of each element in the dataset were calculated and ranked. The final 212 features were retained after selective filtering of the features according to their importance. And the effective information of PNCC was further extracted by CNN. Finally, the optimized time–frequency domain features and the reduced-dimensional PNCC were combined as fusion features, as shown in Fig. 9.

## Classification

XGBoost, that is, Xtreme Gradient Boosting, is a kind of continuous optimization of the algorithm by continuously adding tree branches. When we complete the training and get k trees, then according to its characteristics, the sum of the scores corresponding to the leaves is the predicted value of the sample. XGBoost is an additive extension to build the objective function by reducing the loss function, using only the decision tree as the base classifier, and using the loss function variation to control the complexity of the tree.

XGBoost is an additive module composed of several base modules. Assuming that the tree model to be trained in the *t*-th iteration is$\;f_{t}\left(x\right)$, then the predicted result of sample i after t iterations ${\hat{y}}_{i}^{\left(t\right)}$ can be expressed as formula 17:

(17) $${\hat{y}}_{i}^{\left(t\right)}=\sum_{k=1}^{t}f_{k}\left(x_{i}\right)={\hat{y}}_{i}^{\left(t-1\right)}+f_{t}\left(x_{i}\right)$$

where $f_{t}\left(x_{i}\right)$ is the model of the t-th tree, and ${\hat{y}}_{i}^{\left(t-1\right)}$ is the prediction result of the (t-1)-th tree, which is a known constant.

Denote the loss after round t as obj, and its calculation formula 18 is:

(18) $$obj^{\left(t\right)}=\sum_{i=1}^{n}l\left(y_{i},{\hat{y}}_{i}^{\left(t\right)}\right)+\sum_{i=1}^{t}\Omega \left(f_{i}\right)=\sum_{i=1}^{n}l\left(y_{i},{\hat{y}}_{i}^{\left(t-1\right)}+f_{t}\left(x_{i}\right)\right)+\Omega \left(f_{t}\right)+{\hat{y}}_{i}^{\left(t-1\right)}$$

Among them, $l(y_{i},{\hat{y}}_{i}^{\left(t\right)})$ is the loss function, $f_{t}\left(x_{i}\right)$ is the tree model required for the t -th training, measuring the predicted value ${\hat{y}}_{i}^{\left(t\right)}$, the deviation between the previous predicted value ${\hat{y}}_{i}^{\left(t-1\right)}$ and the true value$\;y_{i}$, $\Omega (f_{i})$ is the regular term.

Take the loss function MSE defined by formula 19, its sensitivity to larger errors.

(19) $$MSE=\frac{1}{n}\sum_{i=1}^{n}{\left(y_{i}-\hat{y_{i}}\right)}^{2}$$

To apply the Taylor approximation, the objective function is rewritten into the following form by bringing in the MSE loss function.

(20) $$obj^{\left(t\right)}=\sum_{i=1}^{n}{\left(y_{i}-\left({\hat{y}}_{i}^{\left(t-1\right)}+f_{t}\left(x_{i}\right)\right)\right)}^{2}+\sum_{i=1}^{t}\Omega \left(f_{i}\right)=\sum_{i=1}^{n}\left[2\left({\hat{y}}_{i}^{\left(t-1\right)}-y_{i}\right)f_{t}\left(x_{i}\right)+f_{t}{\left(x_{i}\right)}^{2}\right]+\Omega \left(f_{t}\right)+{\hat{y}}_{i}^{\left(t-1\right)}$$

Then, approximated by a second-order Taylor expansion and removing the constant term, the objective function is reduced to a simple sum of quadratic functions that can be easily minimized using a greedy algorithm.

(21) $$obj^{\left(t\right)}=\sum_{i=1}^{n}\left[l\left(y_{i},{\hat{y}}_{i}^{\left(t-1\right)}\right)+g_{i}f_{t}\left(x_{i}\right)+\frac{1}{2}h_{i}f_{t}^{2}\left(x_{i}\right)\right]+\Omega \left(f_{t}\right)+{\hat{y}}_{i}^{\left(t-1\right)}$$

where,$\;g_{i}\;$and $h_{i}$ are defined as:

(22) $$g_{i}\;=\partial_{{\hat{y}}_{i}^{\left(t-1\right)}}l\left(y_{i},{\hat{y}}_{i}^{\left(t-1\right)}\right)$$

(23) $$h_{i}=\partial_{{\hat{y}}_{i}^{\left(t-1\right)}}^{2}l\left(y_{i},{\hat{y}}_{i}^{\left(t-1\right)}\right)$$

When making the t-th prediction, the models from 1 to t-1 are fixed, so $\;l(y_{i},{\hat{y}}_{i}^{\left(t-1\right)})$ is a constant, we remove the constant from the loss function above and the final objective function is obtained, as shown in formula 24:

(24) $$obj^{\left(t\right)}=\sum_{i=1}^{n}\left[g_{i}f_{t}\left(x_{i}\right)+\frac{1}{2}h_{i}f_{t}^{2}\left(x_{i}\right)\right]+\Omega \left(f_{t}\right)$$

where $\Omega (f_{t})$ represents a regularization of penalizes model complexity, resulting in a simpler prediction function. It can be expressed as formula 25:

(25) $$\Omega \left(f_{t}\right)=\gamma T+\frac{1}{2}\lambda \sum_{j=1}^{T}\omega_{j}^{2}$$

where T is the number of leaves in the decision tree, γ and ω are the complexity and vector score of each leaf, respectively, and λ is a constant parameter that measures the degree of penalty.

**Fig. 10:**
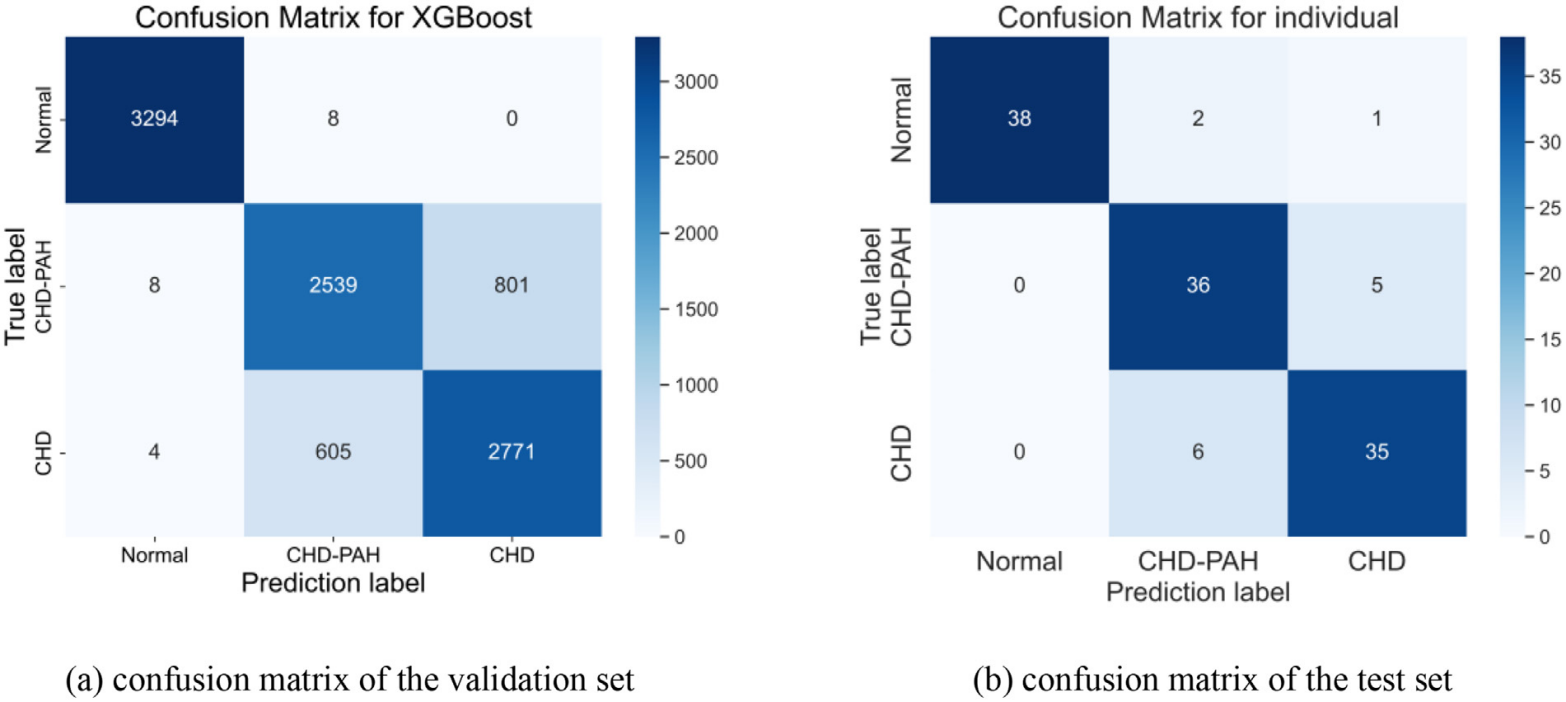
Confusion matrix for three classifications.

XGBoost allows prediction of output variables based on a series of rules arranged in a tree-like structure. Based on the value of the input features, they consist of a series of split points, nodes. The last node is a leaf node that gives us the concrete value of the output variable. The optimal model parameters are obtained in this experiment: the number of trees is 600, the depth of the tree is 7 layers, the minimum weight of leaf nodes is 1, the L1 regularization coefficient is 1, the L2 regularization coefficient is 3, and the learning rate is 0.01. As shown in Table 2, each indicator of the three classifications of this method in the validation set. Fig. 10 shows the confusion matrix obtained by the three classifications of this method.

**Table 2:** Three classifications result in verification set.

Type	Precision	Recall	Accuracy
Normal	0.9963	0.9975	0.8578
CHD-PAH	0.8055	0.7584	
CHD	0.7757	0.8198

## Majority voting algorithm

The problem prototype applied by majority voting calculation finds the most likely multi-number element from the set, which usually overlaps the order of the input conclusion and occupies the majority of all sorted elements.After the first traversal, it is necessary to perform the next traversal to statistically analyze the frequency of the results of the first algorithm traversal, and to determine whether it is a mode or not. In fact, each 20-second heart sound signal recorded includes 20 to 33 cardiac cycles. If only the results of a single cardiac cycle are used as the basis, there will often be misjudgments caused by the chance of a single cycle.The classification result of each sample is obtained by majority voting of the classification results of several cardiac cycles of the sample.

Heart sound signals are quasi-periodic, so the difference between each cardiac cycle is minimal. For the same individual, the classification results for each cardiac cycle should theoretically be the same. Because some non-pathological noises will inevitably be caused during the acquisition process, such as breathing sounds, speaking sounds, and sudden abnormal heart sounds caused by sudden coughing, these factors will cause the classifier to misjudge. In order to reduce the influence of classifier misjudgment on the final classification result, a majority voting algorithm is used to determine the final classification result of the individual. The majority voting algorithm is as follows:

**Table:** 

Initialize an element m and a counter I, with *i* = 0, *m* = 0
For each element x of the input sequence:
If i=0, then assign m=xand i=1
else ifm=x, then assigni=i+1
else assign i=i−1
Return m

In addition, Table 3 shows the indicators obtained from the three classification test sets using this method. The accuracy rate obtained is 88.61%.

**Table 3:** Three classifications result for individual after the majority vote in test set.

Type	Precision	Recall	Accuracy
Normal	0.99	0.9268	0.8861
CHD-PAH	0.8571	0.8780	
CHD	0.8536	0.8536	

## Discussion and conclusion

The features after fusion of time-domain features, frequency-domain features and depth features can represent pathological features. Most voting algorithms can be used to select the best classification result to improve the performance of the algorithm. XGBoost is a better classifier for cases where the number of samples is not too large. The algorithm achieved an accuracy of 87.80% in the dichotomous test set and 88.61% in the trichotomous test set. Compared with existing studies, the method proposed in this paper has good prospects for application in non-invasive CHD-PAH machine-assisted diagnosis.

## CRediT author statement

**Pengyue Ma:** Methodology, Writing Original draft preparation **Bingbing Ge:** Methodology, Software **Hongbo Yang**: Data Collection **Jiahua Pan:** Data curation, Clinical director **Tao Guo:**Supervision **Weilian Wang:** Writing-Reviewing and Editing, **Corresponding author.**

## Declaration of Competing Interest

The authors declare that they have no known competing financial interests or personal relationships that could have appeared to influence the work reported in this paper.

## Data Availability

The authors do not have permission to share data.

